# Bis(acetato-κ^2^ *O*,*O*′)bis­(3,5-dimethyl-1*H*-pyrazole-κ*N* ^2^)copper(II)

**DOI:** 10.1107/S1600536809019400

**Published:** 2009-05-29

**Authors:** Yuliya M. Davydenko, Igor O. Fritsky, Vadim O. Pavlenko, Franc Meyer, Sebastian Dechert

**Affiliations:** a National Taras Shevchenko University, Department of Chemistry, Volodymyrska Street 64, 01601 Kiev, Ukraine; b Institut für Anorganische Chemie, Universität Göttingen, Tammannstrasse 4, 37077 Göttingen, Germany

## Abstract

In the title compound, [Cu(C_2_H_3_O_2_)_2_(C_5_H_8_N_2_)_2_], the Cu^II^ atom has a distorted tetra­gonal–bipyramidal geometry, with the equatorial plane formed by two N atoms belonging to two 3,5-dimethyl-1*H*-pyrazole ligands and two O atoms from two acetate anions. The second O atoms of the acetate groups provide elongated Cu—O axial contacts, so that the acetates appear to be coordinated in a pseudo-chelate fashion. The pyrazole ligands are situated in *cis* positions with respect to each other. In the crystal structure, mol­ecules are linked through inter­molecular N—H⋯O hydrogen bonds, forming a one-dimensional chain.

## Related literature

For properties and applications of 1*H*-pyrazole and its 3,5-substituted derivatives, see: Fritsky *et al.* (1993 ▶, 1994*a* ▶,*b* ▶); Halcrow (2001 ▶); Jain *et al.* (2004 ▶); Krämer (1999 ▶); Krämer *et al.* (2002 ▶); Raptis *et al.* (1999 ▶); Seredyuk *et al.* (2007 ▶); Skopenko *et al.* (1990 ▶). For related compounds, see: Barooah *et al.* (2006 ▶); Deka *et al.* (2006 ▶); Karmakar *et al.* (2007 ▶); Porai-Koshits (1980 ▶); Pradeep *et al.* (2006 ▶).
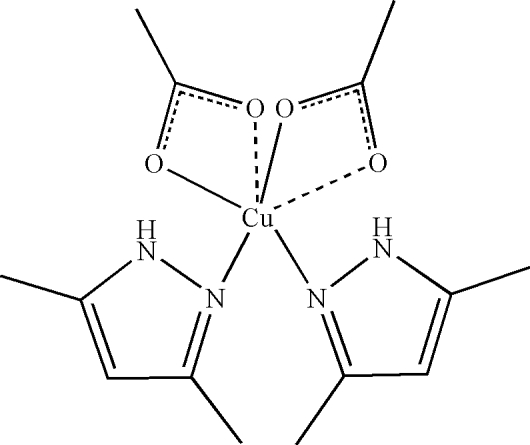


## Experimental

### 

#### Crystal data

[Cu(C_2_H_3_O_2_)_2_(C_5_H_8_N_2_)_2_]
*M* *_r_* = 373.90
Triclinic, 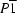

*a* = 9.2861 (11) Å
*b* = 10.1684 (12) Å
*c* = 10.3139 (13) Å
α = 110.755 (9)°
β = 100.901 (10)°
γ = 99.383 (9)°
*V* = 865.7 (2) Å^3^
*Z* = 2
Mo *K*α radiation
μ = 1.29 mm^−1^
*T* = 133 K
0.50 × 0.08 × 0.07 mm

#### Data collection

Stoe IPDSII diffractometer
Absorption correction: numerical (*X-RED*; Stoe & Cie, 2002 ▶) *T*_min_ = 0.790, *T*_max_ = 0.935
7882 measured reflections
3713 independent reflections
3129 reflections with *I* > 2σ(*I*)
*R*_int_ = 0.029

#### Refinement

*R*[*F* ^2^ > 2σ(*F* ^2^)] = 0.032
*wR*(*F* ^2^) = 0.071
*S* = 1.02
3713 reflections
222 parameters
H atoms treated by a mixture of independent and constrained refinement
Δρ_max_ = 0.29 e Å^−3^
Δρ_min_ = −0.66 e Å^−3^

### 

Data collection: *X-AREA* (Stoe & Cie, 2002 ▶); cell refinement: *X-AREA*; data reduction: *X-RED* (Stoe & Cie, 2002 ▶); program(s) used to solve structure: *SHELXS97* (Sheldrick, 2008 ▶); program(s) used to refine structure: *SHELXL97* (Sheldrick, 2008 ▶); molecular graphics: *DIAMOND* (Brandenburg, 1999 ▶); software used to prepare material for publication: *SHELXL97*.

## Supplementary Material

Crystal structure: contains datablocks I, global. DOI: 10.1107/S1600536809019400/hy2196sup1.cif

Structure factors: contains datablocks I. DOI: 10.1107/S1600536809019400/hy2196Isup2.hkl

Additional supplementary materials:  crystallographic information; 3D view; checkCIF report

## Figures and Tables

**Table 1:** Selected bond lengths (Å)

Cu1—N1	1.9851 (18)
Cu1—N3	1.9925 (16)
Cu1—O1	1.9909 (15)
Cu1—O2	2.4774 (18)
Cu1—O3	2.0045 (14)
Cu1—O4	2.4603 (16)

**Table 2:** Hydrogen-bond geometry (Å, °)

*D*—H⋯*A*	*D*—H	H⋯*A*	*D*⋯*A*	*D*—H⋯*A*
N2—H2⋯O4^i^	0.82 (3)	1.92 (3)	2.726 (3)	166 (3)
N4—H4⋯O2^ii^	0.87 (3)	1.91 (3)	2.732 (2)	157 (2)

Symmetry codes: (i) 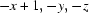; (ii) 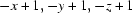.

**Fig. 1.**
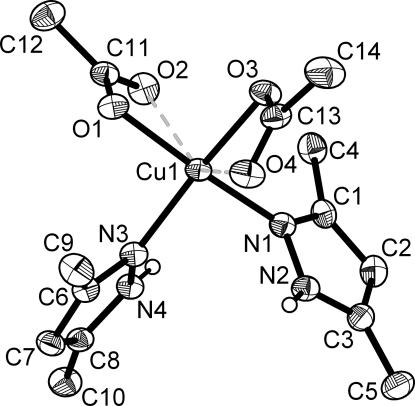
The molecular structure of the title compound. Displacement ellipsoids are drawn at the 50% probability level. The dashed grey lines represent the elongated axial Cu—O bonds.

**Fig. 2.**
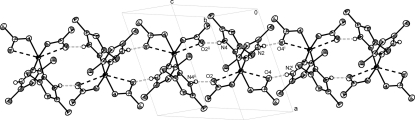
A crystal packing diagram of the title compound, showing the intermolecular hydrogen bonds as underlined dotted and shaded grey lines, which link the molecules into a one-dimensional chain. The dashed black lines represent the axial Cu—O bonds. [Symmetry codes: (i) 1 - x, -y, -z; (ii) 1 - x, 1 - y, 1 - z.]

## supplementary crystallographic information

### Comment

1*H*-Pyrazole and its 3,5-substituted derivatives have been widely used as bridging ligands in molecular magnetism and supramolecular chemistry because of their marked tendency to form high nuclearity species exhibiting specific magnetic properties (Krämer *et al.*, 2002; Seredyuk *et al.*, 2007). Copper complexes containing pyrazole-based ligands are of particular interest in bioinorganic chemistry, as they can be used as models for the active sites in copper proteins like hemocyanine and tyrosinase (Krämer, 1999; Raptis *et al.*, 1999). In addition, copper carboxylates are important in biology and also in basic inorganic chemistry (Halcrow, 2001; Jain *et al.*, 2004). The carboxylates show a large variety of coordination modes, which can lead to the formation of different assemblies, including supramolecular coordination polymers or metal–organic frameworks (Fritsky *et al.*, 1993, 1994*a*,*b*; Skopenko *et al.*, 1990). A large number of copper(II) carboxylates with flexible connection are reported (Barooah *et al.*, 2006; Pradeep *et al.*, 2006). Total use of 1*H*-pyrazole derivatives and carboxylates can lead to the formation of mononuclear complexes with vacant donor atoms, which can be use as building blocks for the preparation of polynuclear complexes or coordination polymers.

The title compound is a mononuclear complex (Fig. 1), which consists of a Cu^II^ ion as the central atom possessing a Jahn–Teller distorted tetragonal–bipyramidal environment. The four equatorial positions are occupied by two N atoms belonging to two monodentately coordinated 3,5-dimethyl-1*H*-pyrazole molecules [Cu—N = 1.9851 (18) and 1.9925 (16) Å] and two O atoms from the acetate anions [Cu—O = 1.9909 (15) and 2.0045 (14) Å]. The other two O atoms of the acetate anions occupy the axial positions [Cu—O = 2.4603 (16) and 2.4774 (18) Å] (Table 1). Each sort of ligands (3,5-dimethyl-1*H*-pyrazole and carboxylate) in the coordination sphere of central Cu^II^ is *cis*-oriented with respect to each other. According to the carboxylate coordination criteria (Poray-Coshits, 1980), the acetate anions of the title compound coordinate in a pseudo-chelate mode, forming a four-membered chelate ring. The specific chelation of the above mentioned acetate anions, when one of the two bonds always resides in the equatorial position and second bond occupies the axial position of tetragonal–bipyramidal environment, was found in many Cu^II^ compounds (Deka *et al.*, 2006; Karmakar *et al.*, 2007). The Cu—O equatorial distances varying in the range of 1.970 and 1.974 Å are somewhat shorter than those in the title compound. The axial Cu—O bond lengths in the title compound are less than 2.685 Å (Karmakar *et al.*, 2007), but longer than 2.281 Å reported by Deka *et al.* (2006). In addition, asymmetric chelation of the acetate anions shows up in inequivalence of the two C—O bonds. Thus, C—O distances adjacent to the elongated axial Cu—O bonds [C13—O4 = 1.250 (3) and C11—O2 = 1.256 (2) Å] are a bit shorter than those in the equatorial plane [C13—O3 = 1.262 (3) and C11—O1 = 1.266 (3) Å]. The values of the angles around the central atom deviate from ideal tetragonal–bipyramidal geometry.

In the crystal packing (Fig. 2), the complex molecules are connected through intermolecular N—H···O hydrogen bonds into a one-dimensional linear chain. The hydrogen bonds in the structure are of two types, with distances N2···O4 = 2.726 (3) and N4···O2 = 2.732 (2)Å (Table 2). Each couple of hydrogen bonds of one type takes part in forming the different ten-membered cycles. Due to this crystal packing, the shortest intra-chain Cu···Cu separations are 6.018 and 6.123 Å.

### Experimental

The title complex was synthesized by a direct method at free access of air oxygen. The mixture of 3,5-dimethyl-1*H*-pyrazole (0.96 g, 0.01 mol), ammonium acetate (0.77 g, 0.01 mol) in dimethylsulfoxide solution (15 ml) was stirred with copper powder (0.64 g, 0.01 mol) at ambient temperature until dissolved. The resulting dark-green solution was filtered and the filtrate was left to stand at room temperature for crystallization in air. Slow evaporation yielded green crystals of the title complex suitable for X-ray analysis in 5 d.

### Refinement

H atoms bound to C atoms were positioned geometrically and refined as riding atoms, with C—H = 0.93 (CH) and 0.96 (CH_3_) Å and with *U*_iso_(H) = 0.08 Å^2^. H atoms bound to N atoms were located on a difference Fourier map and refined isotropically.

### Crystal data

**Table:**

[Cu(C_2_H_3_O_2_)_2_(C_5_H_8_N_2_)_2_]	*Z* = 2
*M**_r_* = 373.90	*F*(000) = 390
Triclinic, *P*1	*D*_x_ = 1.434 Mg m^−^^3^
Hall symbol: -P 1	Mo *K*α radiation, λ = 0.71073 Å
*a* = 9.2861 (11) Å	Cell parameters from 7882 reflections
*b* = 10.1684 (12) Å	θ = 2.2–27.1°
*c* = 10.3139 (13) Å	µ = 1.29 mm^−^^1^
α = 110.755 (9)°	*T* = 133 K
β = 100.901 (10)°	Needle, blue
γ = 99.383 (9)°	0.50 × 0.08 × 0.07 mm
*V* = 865.7 (2) Å^3^	

### Data collection

**Table:**

Stoe IPDSII diffractometer	3713 independent reflections
Radiation source: fine-focus sealed tube	3129 reflections with *I* > 2σ(*I*)
graphite	*R*_int_ = 0.029
ω scans	θ_max_ = 27.1°, θ_min_ = 2.2°
Absorption correction: numerical (*X-RED*; Stoe & Cie, 2002)	*h* = −11→11
*T*_min_ = 0.790, *T*_max_ = 0.935	*k* = −12→12
7882 measured reflections	*l* = −13→13

### Refinement

**Table:**

Refinement on *F*^2^	Primary atom site location: structure-invariant direct methods
Least-squares matrix: full	Secondary atom site location: difference Fourier map
*R*[*F*^2^ > 2σ(*F*^2^)] = 0.032	Hydrogen site location: inferred from neighbouring sites
*wR*(*F*^2^) = 0.071	H atoms treated by a mixture of independent and constrained refinement
*S* = 1.02	*w* = 1/[σ^2^(*F*_o_^2^) + (0.0405*P*)^2^] where *P* = (*F*_o_^2^ + 2*F*_c_^2^)/3
3713 reflections	(Δ/σ)_max_ < 0.001
222 parameters	Δρ_max_ = 0.29 e Å^−^^3^
0 restraints	Δρ_min_ = −0.66 e Å^−^^3^

### Fractional atomic coordinates and isotropic or equivalent isotropic displacement parameters (Å^2^)

**Table:**

	*x*	*y*	*z*	*U*_iso_*/*U*_eq_	
Cu1	0.59542 (3)	0.26364 (3)	0.28774 (3)	0.01892 (8)	
N1	0.46111 (18)	0.31000 (19)	0.1448 (2)	0.0219 (4)	
N2	0.35711 (19)	0.2059 (2)	0.0274 (2)	0.0236 (4)	
N3	0.41420 (18)	0.17191 (19)	0.3311 (2)	0.0206 (4)	
N4	0.30556 (18)	0.2416 (2)	0.3681 (2)	0.0208 (4)	
O1	0.73397 (15)	0.25272 (16)	0.45427 (17)	0.0241 (3)	
O2	0.69120 (17)	0.47040 (17)	0.52556 (18)	0.0290 (3)	
O3	0.77354 (16)	0.32001 (17)	0.21787 (18)	0.0274 (3)	
O4	0.67255 (17)	0.08587 (17)	0.09809 (18)	0.0313 (4)	
C1	0.4435 (2)	0.4379 (2)	0.1444 (2)	0.0236 (4)	
C2	0.3264 (2)	0.4129 (3)	0.0235 (3)	0.0281 (5)	
H2A	0.2906	0.4827	−0.0025	0.080*	
C3	0.2751 (2)	0.2644 (3)	−0.0486 (2)	0.0264 (5)	
C4	0.5404 (3)	0.5761 (2)	0.2603 (3)	0.0295 (5)	
H4A	0.4975	0.6013	0.3409	0.080*	
H4B	0.5460	0.6519	0.2251	0.080*	
H4C	0.6402	0.5645	0.2900	0.080*	
C5	0.1575 (3)	0.1717 (3)	−0.1863 (3)	0.0361 (6)	
H5A	0.2058	0.1261	−0.2587	0.080*	
H5B	0.1002	0.2310	−0.2170	0.080*	
H5C	0.0909	0.0986	−0.1719	0.080*	
C6	0.3738 (2)	0.0426 (2)	0.3370 (2)	0.0223 (4)	
C7	0.2394 (2)	0.0308 (2)	0.3797 (3)	0.0260 (5)	
H7	0.1875	−0.0481	0.3924	0.080*	
C8	0.1996 (2)	0.1597 (2)	0.3991 (2)	0.0226 (4)	
C9	0.4646 (3)	−0.0668 (3)	0.2981 (3)	0.0331 (5)	
H9A	0.4376	−0.1180	0.1954	0.080*	
H9B	0.4442	−0.1345	0.3417	0.080*	
H9C	0.5705	−0.0183	0.3320	0.080*	
C10	0.0700 (2)	0.2149 (3)	0.4456 (3)	0.0317 (5)	
H10A	0.1082	0.3080	0.5246	0.080*	
H10B	0.0138	0.1479	0.4753	0.080*	
H10C	0.0049	0.2240	0.3668	0.080*	
C11	0.7570 (2)	0.3795 (2)	0.5494 (2)	0.0232 (4)	
C12	0.8678 (3)	0.4198 (3)	0.6921 (3)	0.0344 (5)	
H12A	0.9637	0.4742	0.6944	0.080*	
H12B	0.8803	0.3331	0.7054	0.080*	
H12C	0.8303	0.4777	0.7678	0.080*	
C13	0.7706 (2)	0.1986 (3)	0.1243 (2)	0.0268 (5)	
C14	0.8912 (3)	0.1923 (3)	0.0435 (3)	0.0450 (7)	
H14A	0.9847	0.1944	0.1038	0.080*	
H14B	0.9053	0.2743	0.0174	0.080*	
H14C	0.8601	0.1042	−0.0418	0.080*	
H4	0.319 (3)	0.334 (3)	0.384 (3)	0.023 (6)*	
H2	0.351 (3)	0.118 (3)	0.003 (3)	0.027 (7)*	

### Atomic displacement parameters (Å^2^)

**Table:**

	*U*^11^	*U*^22^	*U*^33^	*U*^12^	*U*^13^	*U*^23^
Cu1	0.01590 (12)	0.01845 (13)	0.02150 (14)	0.00555 (9)	0.00670 (9)	0.00546 (10)
N1	0.0212 (8)	0.0212 (9)	0.0221 (10)	0.0048 (7)	0.0071 (7)	0.0068 (8)
N2	0.0227 (8)	0.0215 (10)	0.0224 (10)	0.0043 (7)	0.0060 (7)	0.0047 (8)
N3	0.0179 (7)	0.0201 (9)	0.0239 (10)	0.0087 (7)	0.0071 (7)	0.0064 (8)
N4	0.0182 (8)	0.0218 (9)	0.0241 (10)	0.0090 (7)	0.0086 (7)	0.0078 (8)
O1	0.0202 (7)	0.0217 (8)	0.0275 (9)	0.0083 (6)	0.0053 (6)	0.0057 (7)
O2	0.0331 (8)	0.0240 (8)	0.0318 (9)	0.0132 (7)	0.0121 (7)	0.0088 (7)
O3	0.0225 (7)	0.0264 (8)	0.0282 (9)	0.0029 (6)	0.0101 (6)	0.0046 (7)
O4	0.0274 (8)	0.0259 (8)	0.0331 (10)	0.0053 (7)	0.0110 (7)	0.0024 (7)
C1	0.0259 (10)	0.0257 (11)	0.0233 (12)	0.0093 (8)	0.0126 (9)	0.0102 (10)
C2	0.0303 (11)	0.0317 (12)	0.0293 (13)	0.0140 (9)	0.0125 (10)	0.0151 (11)
C3	0.0215 (9)	0.0365 (13)	0.0242 (12)	0.0094 (9)	0.0096 (9)	0.0126 (10)
C4	0.0364 (11)	0.0227 (11)	0.0294 (13)	0.0073 (9)	0.0106 (10)	0.0097 (10)
C5	0.0264 (11)	0.0475 (15)	0.0279 (13)	0.0065 (10)	0.0030 (10)	0.0109 (12)
C6	0.0225 (9)	0.0204 (10)	0.0233 (11)	0.0077 (8)	0.0063 (8)	0.0069 (9)
C7	0.0208 (9)	0.0273 (11)	0.0302 (12)	0.0035 (8)	0.0076 (9)	0.0123 (10)
C8	0.0175 (9)	0.0267 (11)	0.0214 (11)	0.0057 (8)	0.0054 (8)	0.0069 (9)
C9	0.0336 (11)	0.0251 (12)	0.0473 (16)	0.0153 (10)	0.0167 (11)	0.0155 (11)
C10	0.0225 (10)	0.0410 (14)	0.0336 (13)	0.0129 (10)	0.0137 (10)	0.0118 (12)
C11	0.0185 (9)	0.0244 (11)	0.0256 (12)	0.0059 (8)	0.0086 (8)	0.0073 (9)
C12	0.0298 (11)	0.0380 (14)	0.0276 (13)	0.0105 (10)	0.0049 (10)	0.0048 (11)
C13	0.0203 (9)	0.0308 (12)	0.0238 (12)	0.0061 (9)	0.0048 (9)	0.0053 (10)
C14	0.0316 (12)	0.0565 (18)	0.0360 (15)	0.0053 (12)	0.0197 (11)	0.0029 (14)

### Geometric parameters (Å, °)

**Table:**

Cu1—N1	1.9851 (18)	C4—H4B	0.9600
Cu1—N3	1.9925 (16)	C4—H4C	0.9600
Cu1—O1	1.9909 (15)	C5—H5A	0.9600
Cu1—O2	2.4774 (18)	C5—H5B	0.9600
Cu1—O3	2.0045 (14)	C5—H5C	0.9600
Cu1—O4	2.4603 (16)	C6—C7	1.402 (3)
N1—C1	1.338 (3)	C6—C9	1.492 (3)
N1—N2	1.355 (3)	C7—C8	1.377 (3)
N2—C3	1.342 (3)	C7—H7	0.9300
N2—H2	0.82 (3)	C8—C10	1.497 (3)
N3—C6	1.333 (3)	C9—H9A	0.9600
N3—N4	1.361 (2)	C9—H9B	0.9600
N4—C8	1.343 (3)	C9—H9C	0.9600
N4—H4	0.87 (3)	C10—H10A	0.9600
O1—C11	1.266 (3)	C10—H10B	0.9600
O2—C11	1.256 (2)	C10—H10C	0.9600
O3—C13	1.262 (3)	C11—C12	1.502 (3)
O4—C13	1.250 (3)	C12—H12A	0.9600
C1—C2	1.405 (3)	C12—H12B	0.9600
C1—C4	1.485 (3)	C12—H12C	0.9600
C2—C3	1.377 (3)	C13—C14	1.513 (3)
C2—H2A	0.9300	C14—H14A	0.9600
C3—C5	1.492 (3)	C14—H14B	0.9600
C4—H4A	0.9600	C14—H14C	0.9600
			
N1—Cu1—O1	170.00 (7)	C3—C5—H5A	109.5
N1—Cu1—N3	89.80 (7)	C3—C5—H5B	109.5
O1—Cu1—N3	91.52 (7)	H5A—C5—H5B	109.5
N1—Cu1—O3	90.43 (7)	C3—C5—H5C	109.5
O1—Cu1—O3	90.04 (6)	H5A—C5—H5C	109.5
N3—Cu1—O3	169.70 (7)	H5B—C5—H5C	109.5
N1—Cu1—O4	91.91 (7)	N3—C6—C7	109.75 (17)
O1—Cu1—O4	96.79 (6)	N3—C6—C9	121.28 (18)
N3—Cu1—O4	111.84 (6)	C7—C6—C9	128.9 (2)
O3—Cu1—O4	57.86 (6)	C8—C7—C6	106.03 (18)
N1—Cu1—O2	112.23 (6)	C8—C7—H7	127.0
O1—Cu1—O2	57.78 (6)	C6—C7—H7	127.0
N3—Cu1—O2	95.42 (7)	N4—C8—C7	106.80 (17)
O3—Cu1—O2	94.03 (6)	N4—C8—C10	121.04 (19)
O4—Cu1—O2	143.81 (5)	C7—C8—C10	132.2 (2)
C1—N1—N2	106.62 (17)	C6—C9—H9A	109.5
C1—N1—Cu1	130.74 (16)	C6—C9—H9B	109.5
N2—N1—Cu1	122.49 (13)	H9A—C9—H9B	109.5
C3—N2—N1	111.34 (19)	C6—C9—H9C	109.5
C3—N2—H2	125.2 (19)	H9A—C9—H9C	109.5
N1—N2—H2	123.2 (18)	H9B—C9—H9C	109.5
C6—N3—N4	106.06 (15)	C8—C10—H10A	109.5
C6—N3—Cu1	131.10 (13)	C8—C10—H10B	109.5
N4—N3—Cu1	122.80 (13)	H10A—C10—H10B	109.5
C8—N4—N3	111.35 (17)	C8—C10—H10C	109.5
C8—N4—H4	127.8 (15)	H10A—C10—H10C	109.5
N3—N4—H4	119.9 (15)	H10B—C10—H10C	109.5
C11—O1—Cu1	101.34 (13)	O2—C11—O1	121.5 (2)
C11—O2—Cu1	79.30 (13)	O2—C11—C12	120.4 (2)
C13—O3—Cu1	100.41 (13)	O1—C11—C12	118.11 (19)
C13—O4—Cu1	79.78 (13)	C11—C12—H12A	109.5
N1—C1—C2	109.0 (2)	C11—C12—H12B	109.5
N1—C1—C4	120.58 (19)	H12A—C12—H12B	109.5
C2—C1—C4	130.46 (19)	C11—C12—H12C	109.5
C3—C2—C1	106.30 (19)	H12A—C12—H12C	109.5
C3—C2—H2A	126.8	H12B—C12—H12C	109.5
C1—C2—H2A	126.8	O4—C13—O3	121.95 (19)
N2—C3—C2	106.8 (2)	O4—C13—C14	120.2 (2)
N2—C3—C5	121.5 (2)	O3—C13—C14	117.8 (2)
C2—C3—C5	131.7 (2)	C13—C14—H14A	109.5
C1—C4—H4A	109.5	C13—C14—H14B	109.5
C1—C4—H4B	109.5	H14A—C14—H14B	109.5
H4A—C4—H4B	109.5	C13—C14—H14C	109.5
C1—C4—H4C	109.5	H14A—C14—H14C	109.5
H4A—C4—H4C	109.5	H14B—C14—H14C	109.5
H4B—C4—H4C	109.5		

### Hydrogen-bond geometry (Å, °)

**Table:**

*D*—H···*A*	*D*—H	H···*A*	*D*···*A*	*D*—H···*A*
N2—H2···O4^i^	0.82 (3)	1.92 (3)	2.726 (3)	166 (3)
N4—H4···O2^ii^	0.87 (3)	1.91 (3)	2.732 (2)	157 (2)

Symmetry codes: (i) −*x*+1, −*y*, −*z*; (ii) −*x*+1, −*y*+1, −*z*+1.
